# Education and Training Needs of Health Care Professionals in the Philippines Encountering Patients with Lung Oligometastatic Cancers

**DOI:** 10.3390/curroncol31120586

**Published:** 2024-12-13

**Authors:** Daphne J. Valmonte, Naa Kwarley Quartey, Fatima Gutierrez, Janel Mendoza, Janet Papadakos, Meredith Giuliani

**Affiliations:** 1 Department of Radiation Oncology, Princess Margaret Cancer Centre, 610 University Ave, Toronto, ON M5G 2M9, Canada; 2 Cancer Education, Princess Margaret Cancer Centre, 610 University Ave, Toronto, ON M5G 2M9, Canada; 3 MakatiMed Wellness Center, 7th Floor, Ayala North Exchange Tower 1 6796 Ayala Avenue, cor Salcedo, Legaspi Village, Makati 1226, Philippinesjanel.medoza@makatimed.net.ph (J.M.); 4 The Institute for Education Research, University Health Network, 222 St. Patrick Street, Toronto, ON M5T 1V4, Canada; 5 The Institute for Health Policy, Management & Evaluation, University of Toronto, 155 College Street, Suite 425, Toronto, ON M5T 3M6, Canada; 6 Department of Radiation Oncology, University of Toronto, 149 College Street, Suite 504, Toronto, ON M5T 1P5, Canada

**Keywords:** lung oligometastasis, stereotactic body radiation therapy (SBRT), radiotherapy, healthcare provider training, low- and middle-income countries (LMICs), clinical oncology curriculum, training needs assessment

## Abstract

This study aimed to examine the education and training needs of health care practitioners (HCPs) in the Philippines who encounter lung oligometastatic cancer patients. Lung oligometastatic disease is among the most common sites for cancer spread and has the most established practices for treating oligometastases. A modified version of the Hennessy–Hicks Training Needs Assessment Questionnaire was administered online to HCPs working in private and public centers in the Philippines. HCPs were recruited via purposive sampling. Twenty-seven HCPs completed the questionnaire (47% response rate). Respondents were mostly female (59%) and between the ages of 30 and 39 years (70%). Three-quarters (74%) were consultants, and most respondents were radiation oncologists (44%) or medical oncologists (30%). Medical oncologists rated Management/Supervisory Tasks (mean = 1.42) as their highest area of training need while radiation oncologists rated Clinical Tasks (mean = 1.30) as their highest training need. Pulmonologists (mean = 0.60) and other specialists (mean = 1.00) rated Administration tasks as their top area of training need. The clinical task-related category was rated the highest need among the continuing medical education topics. This study provides valuable insights for the implementation and advancement of a comprehensive curriculum in clinical oncology, specifically designed to enhance the administrative, clinical, and research capacities of oncologists who encounter oligometastatic lung disease in the Philippines.

## 1. Introduction

Over the next 2 decades, the burden of cancer is estimated to increase by 60%. This increased burden is expected to result in 20 million new cases of cancer and 10 million cancer deaths globally, with the greatest impact on low- and middle-income countries (LMICs) [1]. In the Philippines, an LMIC where nearly 60% of households are of lower socioeconomic status, cancer is the third leading cause of death with lung cancer ranking number one in mortality [2,3]. Barriers to accessing cancer care in LMICs are two-fold: First, health care practitioners (HCPs) have limited specialized training in cancer control as well as few specialists trained to treat rare cancers [3,4]. Second, for rural populations, cancer care is not accessible without long travel distances and associated costs. For both urban and rural patients, the overall costs of cancer treatment are prohibitively expensive [3]. This study is focused on HCP training needs.

Oligometastases is an evolving classification of metastatic cancer referring to the intermediate state between locally advanced and widely metastatic cancers [5]. Although the definition of oligometastasis is evolving, this disease state involves the presence of approximately 3–5 metastases, consistent with clinical trial design and clinical practice [5]. This disease state can be identified either at the time of initial diagnosis (with synchronous metastases) defined as de novo oligometastatic disease, in the recurrent setting (with metachronous metastases), called oligo-recurrent disease, or as an oligo-progressive disease characterized by disease progression in a few sites while undergoing systemic therapy [5].

Since there is no available biomarker for the identification of patients with true oligometastatic disease, diagnosis is determined solely by imaging tests. This, combined with its insidious and diverse presentation as well as the barriers to cancer treatment in the Philippines, and LMICs more broadly, namely, costs to patients and the lack of training for HCPs, presents significant challenges with the management of this disease.

Oligometastatic cancers have been shown to respond well to local ablative treatment via surgery or stereotactic body radiation therapy (SBRT) [5]. With the advent of improved systemic therapy, including innovative treatments such as immunotherapy, a specific subset of stage IV lung or metastatic cancers from other sites are no longer considered terminal but have become chronic diseases that require some form of local therapy [5]. Despite the emerging evidence for the use of SBRT, and the potential cost savings it affords, there is inconsistent training among radiation oncologists to deliver this therapy. Further, the treatment and management of patients with lung oligometastatic disease are inconsistent around the world. Radiation oncology training in LMICs, such as the Philippines, is limited to a 4-year residency compared to developed countries which offer 5-year residency programs. Further, these 5-year programs offer additional training opportunities in the form of fellowships. Among the nine radiation oncology residency training institutions in the Philippines, five have stereotactic radiosurgery capabilities while four have SBRT capabilities, all of which are private. Thus, only half of the current radiation oncology residents in the Philippines will have SBRT as part of their training. Training in patient selection and delivery of SBRT for patients with oligometastatic disease can improve access to care and possibly reduce the costs to the patient by reducing unnecessary travel for care.

The purpose of this study was to understand the educational and training needs of HCPs, namely radiation oncologists, medical oncologists, thoracic surgeons, and respirologists, in the Philippines, encountering lung oligometastatic cancer patients. Specifically, this assessment was intended to identify which skills HCPs lack, determine how important these skills are in managing patients with lung oligometastatic cancer, and understand which skills should be prioritized for future training. Lung oligometastatic disease was the focus of this study as it is among the most common sites for cancer spread and has the most established practices for treating oligometastases. While other HCPs are involved in the management of lung oligometastatic disease, the specialists named above were surveyed as they are responsible for identifying and referring patients with lung oligometastatic disease for appropriate treatment.

## 2. Materials and Methods

### 2.1. Study Design

A cross-sectional study was conducted at Makati Medical Center in Manila, Philippines, from March to May 2023 and employed a self-administered English language, online questionnaire. HCPs who treat patients with lung oligometastatic cancer, including respirologists or pulmonologists (defined as medical doctors who specialize in the diagnosis and treatment of lung disease), medical oncologists, radiation oncologists, and thoraco-vascular surgeons, were invited to complete the survey. This study was approved by the Research Ethics Board at the University Health Network (REB Number 22-6032). Consent to participate in the study was implied by virtue of the participant submitting the questionnaire.

### 2.2. Recruitment and Questionnaire Administration

The study was introduced to HCPs during monthly lung rounds. Interested HCPs provided their email address to a member of the research team who later sent a follow-up email invitation containing a link to the study questionnaire. A modified Dillman-tailored design method approach was used to promote questionnaire completion. The Dillman method is an approach to designing mail and telephone surveys that considers benefits to participants as well as the researcher [6]. The invitation to HCPs to participate in the study included a note that survey findings would be used to inform future training. In addition, two reminder emails were sent to increase the likelihood of completion. The initial email was sent on 5 March 2023, and reminder emails were sent on 20 March 2023 and 28 April 2023 [7]. Participants were given the option to provide their email address in a separate link upon completion of the questionnaire to receive a local gift card as a token of appreciation for their time.

### 2.3. Measure

The online questionnaire consisted of two sections: (1) participant characteristics and (2) training needs. All participants were invited to complete the same questionnaire. The participant characteristics collected included gender, age, job title, specialization, and number of years in practice.

Participant training needs were assessed using a validated questionnaire developed by Hennessy and Hicks [8]. The Hennessy and Hicks questionnaire was developed to identify the training needs of HCPs to help with priority setting. It can be adapted to any context and any profession without compromising its validity and reliability provided that the instruction manual is followed; this questionnaire has also been used in LMICs. According to the Hennessy–Hicks guidance, new items can be included following face validity testing which can include thematic reviews of the literature, testing with a focus group, or individual assessment, to ensure that they are relevant to the topic [8].

The questionnaire was tailored for this study as per guidance from the developers and adapted to the context of lung oligometastatic cancer care to determine how important a specific skill is to the successful delivery of care (rating A) and how well participants feel they can perform this specific skill (rating B). Tasks were grouped into five categories: (1) Research/Audit, (2) Communication/Teamwork, (3) Clinical Tasks, (4) Administration, and (5) Management/Supervisory. Study participants were also invited to indicate specific areas of their job where they would like further training through an open-ended question. The study team removed 6 items from the original measure, which were irrelevant to a cancer healthcare setting, and added 10 items, specific to the context of lung oligometastatic cancer care. The 10 added tasks were categorized into the Clinical Tasks domain. The final tool was pilot-tested with three HCPs from the Philippines. No changes were made to the questionnaire as a result of this pilot test. The study questionnaire is available as Supplementary File S1.

### 2.4. Analysis

Responses were collected using the online software, LimeSurvey version 3.16.1 (GNU General Public License), and analyzed in Microsoft Excel [9]. Descriptive statistics are reported. Mean self-reported importance and performance ratings for each task were computed to identify training needs within each subcategory. Training needs scores were generated by calculating the difference in importance (rating A) and performance (rating B). The higher the score, the greater the training needs. Differences in training needs were compared between participants’ reported job titles and specializations. Responses to the open-ended question regarding other training needs were coded and summarized to identify additional opportunities for training.

## 3. Results

### 3.1. Demographics

Of the 57 HCPs who received the invitation to complete the questionnaire, 27 completed it, with a response rate of 47%. Participant demographics are described in Table 1. Survey respondents were mostly female (59%). Nearly three-quarters of participants were between 30 and 39 years of age (70%) and classified their job titles as consultants (74%). The majority of participants were medical oncologists (30%) or radiation oncologists (44%) and had been practicing for 1 to 5 years (63%) at the time of completing this survey.

### 3.2. Training Needs

**Figure 1:**
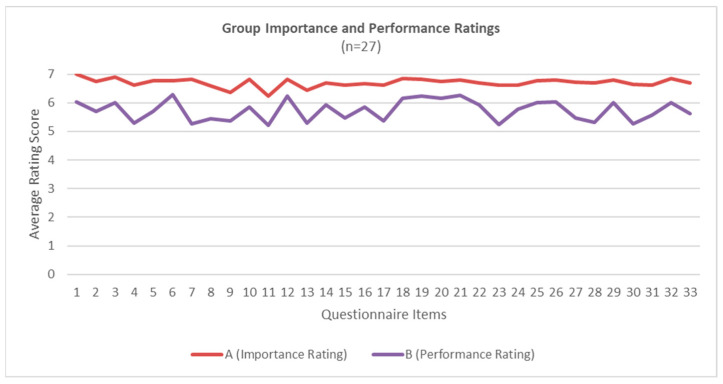
Average importance and performance ratings for all participants.

Respondents provided two ratings for 33 tasks related to the effective performance of their job. Figure 1 summarizes the average importance and performance rating scores for all participants across all questions. Among the study population, establishing a relationship with patients (Q1, X = 7.00) was rated most important while providing feedback to colleagues (Q11, X = 6.22) was ranked lowest. Participants rated communicating with patients face to face as the task they perform the best (Q6, X = 6.30) while providing feedback to colleagues was most often rated as being a task they do not perform well (Q11, X = 5.22).

### 3.3. Training Needs by Specialization

**Figure 2:**
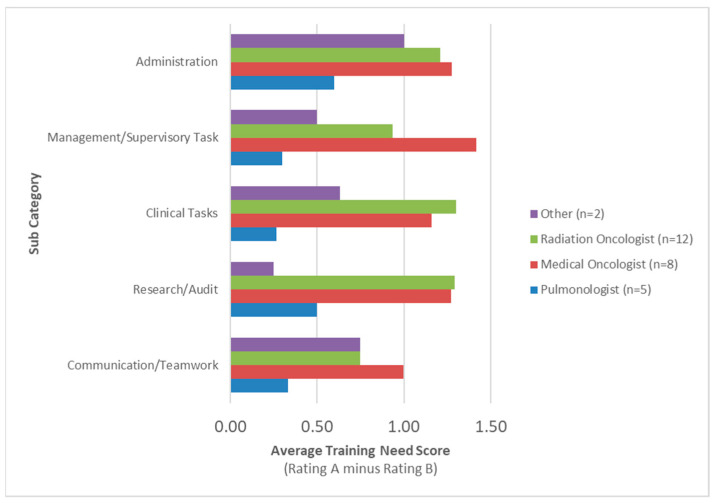
Training needs by specialization.

The top areas of training needs by specialization are summarized in Figure 2 and Table S1. Medical oncologists rated Management/Supervisory Tasks (X = 1.42) as their highest area of training need while radiation oncologists rated Clinical Tasks (X = 1.30) as their highest training need. Pulmonologists (X = 0.60) and other specialists (X = 1.00) rated Administration tasks as their top area of training need. Both medical oncologists (X = 0.75) and radiation oncologists (X = 0.75) rated Communication/Teamwork as their lowest area of training need. Pulmonologists rated Clinical Tasks (X = 0.27) while other specialists (X = 0.25) rated Research/Audit as their lowest area of training need.

### 3.4. Training Needs by Job Title

**Figure 3:**
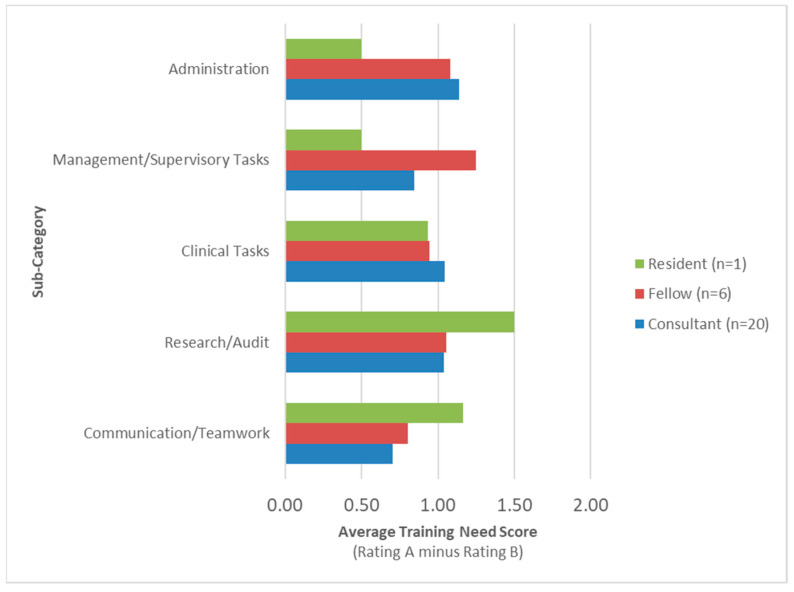
Training needs by job title.

The top area of training needs classified by reported job title is summarized in Figure 3 and Table S2. Consultants rated Administration tasks (X = 1.14) as their highest area of training need while the fellows rated Management/Supervisory Tasks (X = 1.25) as their highest area of training need. The only resident to complete the questionnaire rated Research/Audit (X = 1.50) as the highest area of training need and Management/Supervisory Tasks (X = 0.50) as their lowest area of training need. The fellows (X = 0.80) and consultants (X = 0.70) ranked Communication/Teamwork as their lowest area of training need.

### 3.5. Topics for Further Training

A summary of the training topics from the open-ended question is summarized in Table 2. There were 62 suggested topics participants identified for further training. Three of these topics were not relevant to training in oligometastatic disease management as they were focused on clinical skills that are not involved in the treatment of lung oligometastatic cancers. The remaining 59 comments were categorized as follows: Survivorship and Follow-Up Care 14/59 (23.73%), Research 4/59 (6.78%), Communication 6/59 (10.17%), Knowledge to Practice 5/59 (8.47%), Patient Education 1/59 (1.70%), Diagnosis and Workup 11/59 (18.64%), Treatment 14/59 (23.73%), and Administration 4/59 (6.78%).

## 4. Discussion

Our findings support the adoption and development of a structured curriculum in clinical oncology aimed at building the administrative, clinical, and research capacities of specialists encountering lung oligometastatic cancers in the Philippines, and very likely, other LMICs. Overall, the responses show significant interest among participants in continuing medical education (CME) on the management and care of patients with oligometastatic disease. The topics named below can serve as a framework for future continuing medical education (CME) sessions which can be leveraged to strengthen the practices of HCPs for improved lung oligometastatic cancer care across the globe.

### 4.1. Management and Supervisory

The Management and Supervisory domain included coping with challenges in the health system and making do with limited resources. In 1991, the Philippine health care system was de-centralized and health decisions made at the national level were transferred to local government units (LGUs). Most LGUs lack the infrastructure and specialized doctors, particularly medical and radiation oncologists, limiting access to the most modern radiation treatment techniques and global standard systemic cancer treatments which are more readily available and concentrated in the National Capital Region [10]. Limited resources in the context of lung oligometastasis treatment include a lack of funding for LGUs which, as a result, places the financial burden for lung oligometastasis treatment on patients [4,10,11]. Currently, there are 50 radiation therapy (RT) centers and 73 megavoltage machines (linear accelerators used in RT) in the country, and less than 25% of these RT centers are in public institutions [12]. This supports Cruz-Lim et al.’s study discussing how SBRT is being underutilized in the Philippines because of several barriers such as reimbursement issues with the national health insurance (PhilHealth) and a lack of training.

### 4.2. Administration

Knowing which modality to use to diagnose lung oligometastasis and organizing surveillance for lung metastatic survivors were tasks that fell under this domain. HCPs involved in the management of patients with oligometastasis require training and opportunities, such as a tumor board, to determine the best workup (e.g., CT or MRI) and to determine which ablative and systemic options might be available to manage the disease. It is a necessity for the Philippine healthcare system to develop mechanisms that result in increased funding, resource allocation, and strengthened diagnostic capabilities [13,14].

### 4.3. Research and Audit

In the Research and Audit domain, staying up to date on lung oligometastasis treatment modalities (e.g., RT, surgery, and chemo- and immunotherapy) was the task associated with the largest training need across all categories (refer to Supplementary File S1). Other tasks that had high training needs scores were applying research results from clinical guidelines into your practice and knowing how to evaluate diagnostic information to determine if a lung mass/es is/are metastatic. The training need for research skills was also evident in another LMIC, Uganda, where HCPs completed a training needs assessment which rated research as the largest training need both overall and among the participants [15]. Furthermore, the majority of respondents recognized their lack of capacity to conduct oncologic research, with four out of fifty-nine comments emphasizing the need for adequate hands-on experience in oncology research and even ongoing clinical trials in the Philippines. Ref. [13] discussed solutions to improve cancer clinical trial participation and application in the Philippines which include supporting locally led trials, the encouragement of international collaboration, and strengthening national trial registries to improve awareness of trials. Mentoring and training may serve as good methods to build research capacities [16].

### 4.4. Continuing Medical Education (CME) and Suggested Topics

As shown in Table 2, there were 59 comments that were coded into eight themes. Open-ended comments reinforce the respondents’ need for training in these categories. Of note, there was incongruence regarding one task: communication with colleagues which the respondents felt most confident about in the first part of the questionnaire but was mentioned as a training need in 10% of the open-ended comments (6/60) for suggested topics. Treatment selection is a fundamental and the most complex part of clinical care provision which requires the expertise and collaboration of a multidisciplinary team of specialists [3,10,13,14]. Learning organizations and/or care systems are especially important for LMICs like the Philippines where the need to optimize available scarce resources and or innovate in the absence of recommended solutions is high.

Invariably, the tasks and suggested topics portion of the questionnaire can serve as the basis for lectures and workshops involving care for lung oligometastatic cancers, pave the way for improvement in the quality of cancer care, and mitigate global disparities in cancer outcomes for Filipino cancer patients. In addition, after the implementation of revised training, the same survey can be administered again to determine whether it meets the needs of HCP learners.

### 4.5. Limitations

This study had a small sample size and only one medical resident participant. Thus, the generalizability of results is limited as there was only one resident at the time of questionnaire administration. Although not generalizable, the study can shed some light on curriculum topics to improve training and education for HCPs in the Philippines, and other LMICs, where better support can be provided for the management of rare cancers.

## 5. Conclusions

The Philippines and other LMICs face significant challenges in effectively managing lung oligometastatic disease due to limited healthcare access, a shortage of trained practitioners, and various barriers to cancer care. Additional clinical training to make well-informed decisions regarding diagnostic methods and treatment strategies for patients is needed as well as further training in clinical research and application to ensure that the latest advancements in the field can be effectively utilized. Addressing broader challenges, such as the limited availability of healthcare resources and the high costs associated with treatment, may require the active involvement of national institutions on a larger scale.

Given the outcomes of this study, it is evident that the adoption and advancement of a comprehensive curriculum in clinical oncology is essential for enhancing the administrative, clinical, and research capacity of oncologists who encounter patients with lung oligometastatic cancers in the Philippines.

## Figures and Tables

**Table 1:** Demographic characteristics of survey respondents.

Characteristics of Healthcare Professionals (*n* = 27)		
	*n*	%
Gender		
Female	16	59
Male	11	41
Age, years		
20–29	1	4
30–39	19	70
40–49	3	11
50–59	3	11
>60	1	4
Job Type		
Consultant	20	74
Fellow	6	22
Resident	1	4
Specialization		
Pulmonologist	5	19
Medical Oncologist	8	30
Radiation Oncologist	12	44
Other *	2	7
Years in Practice		
<1	2	7
1–5	17	63
6–10	3	11
11–20	3	11
21–30	2	7

* Other specialists are radiologists and nephrologists.

**Table 2:** Participants’ suggested areas for further training.

Theme	Definition	Subcategory	Suggested Topics	Count	%
Survivorship/Follow-Up Care	Includes follow-up care, surveillance for recurrences after SBRT, and handling late toxicities of RT	Clinical Tasks	• Survivorship care plans • Detection of recurrences	14	23.73
Research	Includes research journals and future directions for research including clinical trials	Research	• Understanding clinical trials • Applying evidence into practice	4	6.78
Communication	Includes multidisciplinary team meetings and collaborative cancer care	Communication/Teamwork	• Improving multidisciplinary team communication	6	10.17
Knowledge to Practice	Includes recent trends and global guidelines for lung SBRT and guidelines	Clinical Tasks	• Updates to practice guidelines	5	8.47
Patient Education	Includes patient resources or how to talk to patients regarding their cancer care journey	Communication/Teamwork	• Improved education and information provision for patients and families	1	1.70
Diagnosis and Workup	Includes how to diagnose oligometastases in patients as well as diagnostic workup	Clinical Tasks	• Management of oligometastases	11	18.64
Treatment	Includes SBRT technicalities and updates on treatment management for specific cancers	Clinical Tasks	• SBRT training • Updates on treatments for specific cancers	14	23.73
Administration	Includes logistics, policies, and protocols	Administration	• Systems improvements	4	6.78

## Data Availability

The original contributions presented in this study are included in the article/Supplementary Materials. Further inquiries can be directed to the corresponding author(s).

## Supplementary Materials

The following supporting information can be downloaded at https://www.mdpi.com/article/10.3390/curroncol31120586/s1: Table S1: Training needs by specialization; Table S2: Training needs by job title. File S1: Additional File 1.
