# Supplementary material for: Education and Training Needs of Health Care Professionals in the Philippines Encountering Patients with Lung Oligometastatic Cancers
Source: Curr Oncol. 2024 Dec 13;31(12):7950–9. doi: 10.3390/curroncol31120586 (PMC11674281; doi:10.3390/curroncol31120586)
Supplement: Supplementary file 1 [file curroncol-31-00586-s001.zip › curroncol-3316028-supplementary.pdf]

Additional File S1

S1 The Subordinate Categories

**Categories**	**The Hennessy Hicks Training Needs Assessment**	**Educational and Training Needs Assessment for HCPs Encountering Lung Oligometastatic Cancers (Pulmonologists, Medical Oncologists, Thoracovascular Surgeons)**
communication/teamwork	1. Establishing a relationship with patients	1. Establishing a relationship with patients
administration	2. Doing paperwork and/or routine data inputting	REMOVED
research/audit	3.Critically evaluating published research	2. Knowing how to evaluate diagnostic information to determine if a lung mass/es is/are metastatic
management/supervisory task	4. Appraising your own performance	REMOVED
communication/teamwork	5. Getting on with your colleagues	3. Communicating with cancer specialists.
research/audit	6. Interpreting your own research findings	4. Interpreting diagnostic images of suspicious lung mass/es
research/audit	7. Applying research results to your own practice	5. Applying research results from clinical guidelines into your practice
communication/teamwork	8. Communicating with patients face-to-face	6. Communicating with patients face-to-face
research/audit	9. Identifying viable research topics	7. Staying up to date on lung oligometastasis treatment modalities (e.g., RT, surgery, chemo and immunotherapy)
clinical tasks	10. Treating patients	8. Understanding patient's pathway of care for investigation of lung masses
management/supervisory task	11. Introducing new ideas at work	9. Introducing new ideas at work
clinical tasks	12.Accessing relevant literature for your clinical work	10. Having access to up-to-date clinical treatment guidelines
communication/teamwork	13. Providing feedback to colleagues	11. Providing feedback to colleagues
communication/teamwork	14. Giving information to patients and/or carers	12. Giving information to patients and/or carers

research/audit	15. Statistically analyzing your own data	REMOVED
management/supervisory task	16. Showing colleagues and/or students how to do things	13. Showing colleagues/supervising trainees on how to manage survivorship and follow-up care of lung oligometastatic patients
clinical tasks	17. Planning and organizing an individual patient’s care	14. Planning and organizing an individual patient's care
clinical tasks	18. Evaluating patients’ psychological and social needs	15. Evaluating patients' psychological and social needs
management/supervisory task	19. Organizing your own time effectively	REMOVED
administration	20. Using technical equipment, including computers	16. Knowing what imaging should be obtained when diagnosing lung oligometastasis
research/audit	21. Writing reports of your research studies	REMOVED
clinical tasks	22. Undertaking health promotion studies	17. Promoting routine follow up studies for lung oligometastasis survivors
management/supervisory task	23. Making do with limited resources	18. Making do with limited resources
clinical tasks	24. Assessing patients’ clinical needs	19. Assessing patients’ clinical needs
research/audit	25. Collecting and collating relevant research information	20. Appropriate investigation and work up for suspicion and initial presentation
research/audit	26. Designing a research study	REMOVED
communication/teamwork	27. Working as a member of a team	21. Working as a member of a team
research/audit	28. Accessing research resources (e.g. time, money, information, equipment)	22. Accessing relevant resources and knowing who to call with questions relating to lung metastasis care
administration	29. Undertaking administrative activities	23. Organizing surveillance for lung metastasis survivors
management/supervisory task	30. Personally coping with change in the health service	24. Coping with challenges in the healthcare system
clinical tasks		25. Being aware of common clinical presentations of lung metastasis
clinical tasks		26. Knowing where to obtain knowledge about whether a suspicious mass could be lung metastasis

clinical tasks		27. Being aware of long-term consequences that lung metastasis survivors face
clinical tasks		28. Identifying gaps in knowledge regarding lung metastatic patient follow-up care
clinical tasks		29. Discussing symptom and pain management with patients
clinical tasks		30. Discussing survivorship care plans with patients
clinical tasks		31. Discussing recurrence with patients
clinical tasks		32. Knowing when to refer potential lung oligometastatic patients to multi-disciplinary team
clinical tasks		33. Knowing when to biopsy an unusual mass to confirm lung metastasis

S2 Final Study Questionnaire

THE HENNESSY-HICKS ASSESSMENT OF TRAINING NEEDS
(Adapted for Health Care Providers encountering Lung Oligometastatic Cancers)

DEMOGRAPHICS

The study team is interested in knowing about you. Your answers will be used to describe who completed this survey. Answer the questions below:

Job Title	❑ Resident<br>❑ Fellow<br>❑ Consultant
Gender	❑ Male<br>❑ Female
Age	❑ <30 years<br>❑ 30-39 years<br>❑ 40-49 years<br>❑ 50-59 years<br>❑ 60 years and above
Subspecialty	❑ Pulmonologist<br>❑ Medical Oncologist<br>❑ Radiation Oncologist<br>❑ Surgeon<br>❑ Other subspecialty, please indicate: __________
Years in practice	❑ Less than one year<br>❑ 1-5 years<br>❑ 6-10 years<br>❑ 11-20 years<br>❑ 21-30 years

INSTRUCTIONS FOR COMPLETION:

This questionnaire comprises two sections that are to do with your training needs. Please answer all the questions as honestly as possible to enable us to compile a complete picture of your training

requirements. Each section is prefaced by instructions for completion. Please read and follow these carefully.

SECTION S1: Training Needs

In order to perform your job effectively you need relevant skills. You will see listed below a range of skilled activities many of which you undertake in performing your job. Look at each of these activities and then rate each one by writing the appropriate number in the box. The first rating (A is concerned with how important the activity is to the successful performance or your job; the second rating (B is concerned with how well you currently perform that activity.

A: How important is this activity to the successful performance of your job?

Rating of 1-7: not at all important = 1; very important = 7

B: How well do you consider that you currently perform this activity?

Rating of 1-7: not well = 1; very well = 7

	**A**	**B**
1. Establishing a relationship with patients		
2. Knowing how to evaluate diagnostic information to determine if a lung mass/es is/are metastatic		
3. Communicating with cancer specialists.		
4. Interpreting diagnostic images of suspicious lung mass/es		
5. Applying research results from clinical guidelines into your practice		
6. Communicating with patients face-to-face		
7. Staying up to date on lung oligometastasis treatment modalities (e.g., RT, surgery, chemo and immunotherapy)		
8. Understanding patient's pathway of care for investigation of lung masses		
9. Introducing new ideas at work		
10. Having access to up-to-date clinical treatment guidelines		
11. Providing feedback to colleagues		
12. Giving information to patients and/or carers		

13. Showing colleagues/supervising trainees on how to manage survivorship and follow-up care of lung oligometastatic patients		
14. Planning and organizing an individual patient's care		
15. Evaluating patients' psychological and social needs		
16. Knowing what imaging should be obtained when diagnosing lung oligometastasis		
17. Promoting routine follow up studies for lung oligometastasis survivors		
18. Making do with limited resources		
19. Assessing patients' clinical needs		
20. Appropriate investigation and work up for suspicion and initial presentation		
21. Working as a member of a team		
22. Accessing relevant resources and knowing who to call with questions relating to lung metastasis care		
23. Organizing surveillance for lung metastasis survivors		
24. Coping with challenges in the healthcare system		
25. Being aware of common clinical presentations of lung metastasis		
26. Knowing where to obtain knowledge about whether a suspicious mass could be lung metastasis		
27. Being aware of long-term consequences that lung metastasis survivors face		
28. Identifying gaps in knowledge regarding lung metastatic patient follow-up care understanding		
29. Discussing symptom and pain management with patients		
30. Discussing survivorship care plans with patients		
31. Discussing recurrence with patients		
32. Knowing when to refer potential lung oligometastatic patients to multi-disciplinary team		
33. Knowing when to biopsy an unusual mass to confirm lung metastasis		

SECTION S2: Specific Training Needs

Please specify the areas of your job in which you would like to receive further training or instruction. List these in order of importance:

1.

2.

3.

4.

5.

6.

7.

8.

9.

10.

Table S1 Training Needs by Specialization

Subcategory	Q#	Task	Specialization														
			All HCPs (n=27)			Radiation Oncologist (n=12)			Medical Oncologist (n=8)			Pulmonologist (n=5)			Other (n=2)**		
			Rating A (mean)	Rating B (mean)	Training Need*** (mean)	Rating A (mean)	Rating B (mean)	Training Need*** (mean)	Rating A (mean)	Rating B (mean)	Training Need*** (mean)	Rating A (mean)	Rating B (mean)	Training Need*** (mean)	Rating A (mean)	Rating B (mean)	Training Need*** (mean)
Administration	16	Knowing what imaging should be obtained when diagnosing lung oligometastasis	6.67	5.85	0.81	6.50	5.83	0.67	6.88	5.75	1.13	6.60	6.00	0.60	7.00	6.00	1.00
	23	Organizing surveillance for lung metastasis survivors	6.62	5.23	1.38	6.42	4.67	1.75	7.00	5.57	1.43	6.80	6.20	0.60	6.00	5.00	1.00
Clinical Tasks	8	Understanding patient's pathway of care for investigation of lung masses	6.59	5.44	1.15	6.42	4.92	1.50	6.75	5.50	1.25	6.80	6.40	0.40	6.50	6.00	0.50
	10	Having access to up-to-date clinical treatment guidelines	6.81	5.85	0.96	6.83	5.50	1.33	7.00	6.25	0.75	6.40	6.00	0.40	7.00	6.00	1.00
	14	Planning and organizing an individual patient's care	6.70	5.93	0.78	6.58	5.67	0.92	6.88	5.88	1.00	7.00	6.80	0.20	6.00	5.50	0.50
	15	Evaluating patients' psychological and social needs	6.63	5.48	1.15	6.50	5.17	1.33	6.75	5.25	1.50	6.60	6.20	0.40	7.00	6.50	0.50
	17	Promoting routine follow up studies for lung oligometastasis survivors	6.63	5.37	1.26	6.33	4.92	1.42	7.00	5.38	1.63	6.80	6.20	0.60	6.50	6.00	0.50
	19	Assessing patients’ clinical needs	6.81	6.22	0.59	6.67	6.17	0.50	7.00	5.88	1.13	6.80	6.80	0.00	7.00	6.50	0.50
	25*	Being aware of common clinical presentations of lung metastasis	6.77	6.00	0.77	6.58	5.58	1.00	7.00	6.00	1.00	6.80	6.80	0.00	7.00	6.50	0.50
	26*	Knowing where to obtain knowledge about whether a suspicious mass could be lung metastasis	6.81	6.04	0.77	6.67	5.75	0.92	7.00	6.14	0.86	6.80	6.60	0.20	7.00	6.00	1.00
	27*	Being aware of long-term consequences that lung metastasis survivors face	6.73	5.46	1.27	6.58	4.83	1.75	7.00	5.71	1.29	6.80	6.40	0.40	6.50	6.00	0.50
	28*	Identifying gaps in knowledge regarding lung metastatic patient	6.69	5.31	1.38	6.42	4.67	1.75	7.00	5.43	1.57	6.80	6.40	0.40	7.00	6.00	1.00

		follow-up care understanding															
	29*	Discussing symptom and pain management with patients	6.81	6.00	0.81	6.83	5.92	0.92	7.00	5.86	1.14	7.00	6.80	0.20	5.50	5.00	0.50
	30*	Discussing survivorship care plans with patients	6.65	5.27	1.38	6.67	4.67	2.00	7.00	5.57	1.43	6.80	6.60	0.20	5.00	4.50	0.50
	31*	Discussing recurrence with patients	6.62	5.58	1.04	6.67	5.50	1.17	6.86	5.57	1.29	6.80	6.40	0.40	5.00	4.00	1.00
	32*	Knowing when to refer potential lung oligometastatic patients to multi-disciplinary team	6.85	6.00	0.85	6.75	5.42	1.33	7.00	6.29	0.71	6.80	6.60	0.20	7.00	7.00	0.00
	33*	Knowing when to biopsy an unusual mass to confirm lung metastasis	6.69	5.62	1.08	6.50	4.83	1.67	6.86	6.00	0.86	6.80	6.80	0.00	7.00	6.00	1.00
Communication / Teamwork	1	Establishing a relationship with patients	7.00	6.04	0.96	7.00	5.83	1.17	7.00	6.00	1.00	7.00	6.40	0.60	7.00	6.50	0.50
	3	Communicating with cancer specialists	6.89	6.00	0.89	6.75	5.83	0.92	7.00	5.63	1.38	7.00	6.80	0.20	7.00	6.50	0.50
	6	Communicating with patients face-to-face	6.78	6.30	0.48	6.58	6.25	0.33	7.00	6.13	0.88	6.80	6.80	0.00	7.00	6.00	1.00
	11	Providing feedback to colleagues	6.22	5.22	1.00	5.83	5.00	0.83	6.50	5.38	1.13	6.60	5.80	0.80	6.50	4.50	2.00
	12	Giving information to patients and/or carers	6.81	6.22	0.59	6.83	6.08	0.75	6.75	6.00	0.75	6.80	6.80	0.00	7.00	6.50	0.50
	21	Working as a member of a team	6.81	6.27	0.54	6.67	6.17	0.50	6.86	6.00	0.86	7.00	6.60	0.40	7.00	7.00	0.00
Management / Supervisory Tasks	9	Introducing new ideas at work	6.37	5.37	1.00	6.17	5.25	0.92	6.50	4.88	1.63	6.60	6.20	0.40	6.50	6.00	0.50
	13	Showing colleagues/supervising trainees on how to manage survivorship and follow-up care of lung oligometastatic patients	6.44	5.30	1.15	6.42	5.08	1.33	6.75	5.38	1.38	6.40	6.00	0.40	5.50	4.50	1.00
	18	Making do with limited resources	6.85	6.15	0.70	6.67	6.00	0.67	7.00	5.75	1.25	7.00	6.80	0.20	7.00	7.00	0.00
	24	Coping with challenges in the healthcare system	6.62	5.77	0.85	6.33	5.50	0.83	6.86	5.43	1.43	6.80	6.60	0.20	7.00	6.50	0.50
Research / Audit	2	Knowing how to evaluate diagnostic information to determine if a lung mass/es is/are metastatic	6.74	5.70	1.04	6.67	5.17	1.50	6.88	5.88	1.00	7.00	6.60	0.40	6.00	6.00	0.00
	4	Interpreting diagnostic images of suspicious lung mass/es	6.63	5.30	1.33	6.42	4.83	1.58	6.75	5.13	1.63	7.00	6.40	0.60	6.50	6.00	0.50
	5	Applying research results from clinical guidelines into your practice	6.78	5.70	1.07	6.67	5.50	1.17	6.88	5.38	1.50	6.80	6.20	0.60	7.00	7.00	0.00

	7	Staying up to date on lung oligometastasis treatment modalities (e.g., Radiation therapy, surgery, chemo and immunotherapy)	6.81	5.26	1.56	6.75	4.83	1.92	7.00	5.38	1.63	6.80	5.80	1.00	6.50	6.00	0.50
	20	Appropriate investigation and work up for suspicion and initial presentation	6.74	6.15	0.59	6.50	5.83	0.67	7.00	6.13	0.88	6.80	6.80	0.00	7.00	6.50	0.50
	22	Accessing relevant resources and knowing who to call with questions relating to lung metastasis care	6.69	5.92	0.77	6.50	5.58	0.92	7.00	6.00	1.00	6.80	6.40	0.40	6.50	6.50	0.00

* Denotes questions added by the study team
** Other specializations include Radiologist and Nephrologist
*** Rating A minus Rating B = training need. Higher the difference in rating, the greater the training need
HCPs = health care professionals

Table S2 Training Needs by Job Title

Subcategory	Q#	Task	Job Title											
			All HCPs (n=27)			Consultant (n=20)			Fellow (n=6)			Resident (n=1)***		
			Rating A (mean)	Rating B (mean)	Training Need** (mean)	Rating A (mean)	Rating B (mean)	Training Need** (mean)	Rating A (mean)	Rating B (mean)	Training Need** (mean)	Rating A	Rating B	Training Need**
Administration	16	Knowing what imaging should be obtained when diagnosing lung oligometastasis	6.67	5.85	0.81	6.70	5.95	0.75	6.67	5.67	1.00	6.00	5.00	1.00
	23	Organizing surveillance for lung metastasis survivors	6.62	5.23	1.38	6.58	5.05	1.53	6.83	5.67	1.17	6.00	6.00	0.00
Clinical Tasks	8	Understanding patient's pathway of care for investigation of lung masses	6.59	5.44	1.15	6.65	5.40	1.25	6.50	5.50	1.00	6.00	6.00	0.00
	10	Having access to up-to-date clinical treatment guidelines	6.81	5.85	0.96	6.75	5.75	1.00	7.00	6.17	0.83	7.00	6.00	1.00
	14	Planning and organizing an individual patient's care	6.70	5.93	0.78	6.70	6.05	0.65	6.67	5.50	1.17	7.00	6.00	1.00
	15	Evaluating patients' psychological and social needs	6.63	5.48	1.15	6.70	5.60	1.10	6.50	5.00	1.50	6.00	6.00	0.00
	17	Promoting routine follow up studies for lung oligometastasis survivors	6.63	5.37	1.26	6.60	5.30	1.30	6.83	5.67	1.17	6.00	5.00	1.00
	19	Assessing patients' clinical needs	6.81	6.22	0.59	6.80	6.35	0.45	6.83	5.83	1.00	7.00	6.00	1.00

	25*	Being aware of common clinical presentations of lung metastasis	6.77	6.00	0.77	6.74	5.95	0.79	6.83	6.17	0.67	7.00	6.00	1.00
	26*	Knowing where to obtain knowledge about whether a suspicious mass could be lung metastasis	6.81	6.04	0.77	6.79	5.95	0.84	6.83	6.33	0.50	7.00	6.00	1.00
	27*	Being aware of long-term consequences that lung metastasis survivors face	6.73	5.46	1.27	6.74	5.32	1.42	6.83	6.00	0.83	6.00	5.00	1.00
	28*	Identifying gaps in knowledge regarding lung metastatic patient follow-up care understanding	6.69	5.31	1.38	6.68	5.11	1.58	6.83	5.83	1.00	6.00	6.00	0.00
	29*	Discussing symptom and pain management with patients	6.81	6.00	0.81	6.79	6.05	0.74	6.83	5.83	1.00	7.00	6.00	1.00
	30*	Discussing survivorship care plans with patients	6.65	5.27	1.38	6.63	5.16	1.47	6.83	5.67	1.17	6.00	5.00	1.00
	31*	Discussing recurrence with patients	6.62	5.58	1.04	6.58	5.58	1.00	6.67	5.67	1.00	7.00	5.00	2.00
	32*	Knowing when to refer potential lung oligometastatic patients to multi-disciplinary team	6.85	6.00	0.85	6.84	5.95	0.89	6.83	6.17	0.67	7.00	6.00	1.00
	33*	Knowing when to biopsy an unusual mass to confirm lung metastasis	6.69	5.62	1.08	6.74	5.58	1.16	6.67	6.00	0.67	6.00	4.00	2.00
Communication / Teamwork	1	Establishing a relationship with patients	7.00	6.04	0.96	7.00	6.10	0.90	7.00	6.00	1.00	7.00	5.00	2.00
	3	Communicating with cancer specialists	6.89	6.00	0.89	6.90	6.10	0.80	6.83	5.67	1.17	7.00	6.00	1.00
	6	Communicating with patients face-to-face	6.78	6.30	0.48	6.75	6.40	0.35	6.83	6.00	0.83	7.00	6.00	1.00
	11	Providing feedback to colleagues	6.22	5.22	1.00	6.25	5.10	1.15	6.17	5.67	0.50	6.00	5.00	1.00
	12	Giving information to patients and/or carers	6.81	6.22	0.59	6.90	6.35	0.55	6.50	5.83	0.67	7.00	6.00	1.00
	21	Working as a member of a team	6.81	6.27	0.54	6.84	6.37	0.47	6.67	6.00	0.67	7.00	6.00	1.00
Management / Supervisory Tasks	9	Introducing new ideas at work	6.37	5.37	1.00	6.50	5.60	0.90	6.00	4.50	1.50	6.00	6.00	0.00
	13	Showing colleagues/supervising trainees on how to manage survivorship and follow-up care of lung oligometastatic patients	6.44	5.30	1.15	6.40	5.25	1.15	6.50	5.33	1.17	7.00	6.00	1.00
	18	Making do with limited resources	6.85	6.15	0.70	6.85	6.25	0.60	6.83	5.83	1.00	7.00	6.00	1.00
	24	Coping with challenges in the healthcare system	6.62	5.77	0.85	6.63	5.89	0.74	6.67	5.33	1.33	6.00	6.00	0.00
Research / Audit	2	Knowing how to evaluate diagnostic information to determine if a lung mass/es is/are metastatic	6.74	5.70	1.04	6.75	5.75	1.00	6.67	5.67	1.00	7.00	5.00	2.00
	4	Interpreting diagnostic images of suspicious lung mass/es	6.63	5.30	1.33	6.70	5.25	1.45	6.50	5.50	1.00	6.00	5.00	1.00
	5	Applying research results from clinical guidelines into your practice	6.78	5.70	1.07	6.80	5.90	0.90	6.67	5.17	1.50	7.00	5.00	2.00

	7	Staying up to date on lung oligometastasis treatment modalities (e.g., Radiation therapy, surgery, chemo and immunotherapy)	6.81	5.26	1.56	6.80	5.20	1.60	6.83	5.50	1.33	7.00	5.00	2.00
	20	Appropriate investigation and work up for suspicion and initial presentation	6.74	6.15	0.59	6.75	6.25	0.50	6.83	6.00	0.83	6.00	5.00	1.00
	22	Accessing relevant resources and knowing who to call with questions relating to lung metastasis care	6.69	5.92	0.77	6.63	5.84	0.79	6.83	6.17	0.67	7.00	6.00	1.00

* Denotes questions added by the study team

** Rating A minus Rating B = training need. Higher the difference in rating, the greater the training need

***Only 1 resident completed the survey; mean sores not calculated

HCPs = health care professional
